# The Diagnostic Value of MRI Pattern Recognition in Distal Myopathies

**DOI:** 10.3389/fneur.2018.00456

**Published:** 2018-06-26

**Authors:** Enrico Bugiardini, Jasper M. Morrow, Sachit Shah, Claire L. Wood, David S. Lynch, Alan M. Pitmann, Mary M. Reilly, Henry Houlden, Emma Matthews, Matt Parton, Michael G. Hanna, Volker Straub, Tarek A. Yousry

**Affiliations:** ^1^MRC Centre for Neuromuscular Diseases, UCL Institute of Neurology and National Hospital for Neurology and Neurosurgery, London, United Kingdom; ^2^Neuroradiological Academic Unit, UCL Institute of Neurology, London, United Kingdom; ^3^Lysholm Department of Neuroradiology, National Hospital for Neurology and Neurosurgery, London, United Kingdom; ^4^John Walton Muscular Dystrophy Research Centre, Institute of Genetic Medicine, Newcastle upon Tyne, United Kingdom; ^5^Department of Molecular Neuroscience, UCL Institute of Neurology, London, United Kingdom

**Keywords:** distal myopathies, muscular dystrophies, MRI pattern, imaging genetics, next generation sequencing

## Abstract

**Objective:** Distal myopathies are a diagnostically challenging group of diseases. We wanted to understand the value of MRI in the current clinical setting and explore the potential for optimizing its clinical application.

**Methods:** We retrospectively audited the diagnostic workup in a distal myopathy patient cohort, reassessing the diagnosis, whilst documenting the usage of MRI. We established a literature based distal myopathies MRI pattern template and assessed its diagnostic utility in terms of sensitivity, specificity, and potential impact on the diagnostic workup.

**Results:** Fifty-five patients were included; in 38 with a comprehensive set of data the diagnostic work-up was audited. The median time from symptoms onset to diagnosis was 12.1 years. The initial genetic diagnostic rate was 39%; 18% were misdiagnosed as neuropathies and 13% as inclusion body myositis (IBM). Based on 21 publications we established a MRI pattern template. Its overall sensitivity (50%) and specificity (32%) were low. However in some diseases (e.g., *MYOT*-related myopathy, *TTN*-HMERF) MRI correctly identified the causative gene. The number of genes suggested by MRI pattern analysis was smaller compared to clinical work up (median 1 vs. 9, *p* < 0.0001) but fewer genes were correctly predicted (5/10 vs. 7/10). MRI analysis ruled out IBM in all cases.

**Conclusion:** In the diagnostic work-up of distal myopathies, MRI is useful in assisting genetic testing and avoiding misdiagnosis (IBM). The overall low sensitivity and specificity limits its generalized use when traditional single gene test methods are applied. However, in the context of next generation sequencing MRI may represent a valuable tool for interpreting complex genetic results.

## Introduction

Distal myopathies are a group of hereditary muscle disorders characterized by weakness of the distal muscles of the upper and more commonly the lower limbs (1). The diagnostic process is particularly complex due to the high number of causative genes and the difficulty in distinguishing patients with distal myopathies from patients with motor neuropathies or muscular dystrophies with distal presentation. In 2009, the 165th ENMC International Workshop on distal myopathies proposed a diagnostic algorithm based on clinical and pathological features, and anterior or posterior lower leg involvement on muscle MRI (1). This approach theoretically reduces the differential diagnosis, however distal myopathies still remain a diagnostically challenging disease group.

The application of muscle imaging is an increasingly recognized tool in the diagnostic workup of muscle disorders and as an outcome measure to quantify disease progression (2, 3). In the diagnostic setting muscle MRI has a dual role. It can guide the biopsy and help to establish the diagnosis based on the recognition of MRI patterns providing useful clues to guide genetic testing. However, the majority of studies described muscle patterns in homogeneous patient cohorts without disease control groups, thus not evaluating the usefulness of the diagnostic application of muscle MRI patterns in clinical practice. The few studies that have evaluated the usefulness of pattern analysis have shown different results depending on the subgroup of diseases analyzed. For example, in limb girdle muscular dystrophy pattern recognition analysis showed low diagnostic yield, whereas in muscular dystrophies with rigidity of the spine the patterns appeared specific and useful (4, 5).

Similarly, in distal myopathies several publications have described a specific pattern of muscle involvement in specific genetic subgroups, but no studies have evaluated the usefulness of MRI pattern recognition in clinical practice.

To understand the value of MRI in the current clinical setting and explore the potential for optimizing its clinical application we retrospectively audited the diagnostic workup in a cohort of patients with distal myopathy, reassessing the diagnosis based on the available clinical and pathological information, whilst documenting the usage of MRI in these patients. We then established a literature based distal myopathies MRI pattern template and assessed its diagnostic utility in terms of its sensitivity and specificity as well as in terms of its potential impact on the diagnostic workup of the patients audited.

## Materials and methods

### Patient selection

In this study we included patients with distal myopathy evaluated at the MRC Centre for Neuromuscular Diseases (UK) that met the following inclusion criteria:

1. First symptoms were of distal weakness or patients had predominantly distal weakness at assessment.
2. The underlying pathogenesis was considered myopathic based on the overall analysis of neurophysiological (electromyography, EMG) or muscle biopsy results. Patients with discordant EMG and muscle biopsy results were included.
3. Either patients had a genetically confirmed distal myopathy [as listed in the classification reported in Udd et al. (6)] or a likely genetic diagnosis (positive family history, or based on slowly progressive presentation and exclusion of likely acquired causes).

Subjects with genetically confirmed myotonic dystrophy type 1 (DM1) or facioscapulohumeral muscular dystrophy (FSHD) were excluded.

This study was performed under the ethical guidelines issued by our institution (University College London Hospitals) for clinical audit studies. The protocol was approved by the Audit Committee at the University College London Hospitals (London, UK). Written informed consent was obtained from all subjects before genetic testing in accordance with the Declaration of Helsinki. Diagnostic facilities at the John Walton Muscular Dystrophy Research Centre are supported by the Rare Diseases Advisory Group Service for Neuromuscular Diseases (NHS England).

### Assessment of diagnostic workup

#### Audited cohort and data collection

We retrospectively audited the overall diagnostic workup of all patients seen between January 2007 and December 2014 that met the inclusion criteria reported above and in whom clinical data were available including age of onset of symptoms, family history, clinical strength assessment by MRC scale (7) and electrophysiological, muscle biopsy and genetic tests if performed. We also assessed the time it took from first evaluation at our center to perform electromyography, first muscle biopsy, second muscle biopsy (if any), muscle MRI and genetic test result. As the first muscle MRI in this cohort was performed in 2006, we considered only the subgroup of patients seen for the first time after 2006 when evaluating the time lag between clinical evaluation and MRI. We finally determined the time it took from the onset of symptoms to a genetic diagnosis.

#### Diagnostic re-evaluation

We re-evaluated the diagnosis of the audited patients based on their clinical and pathological features. For each patient a phenotype was established, taking into account age of disease onset, neurological examination, electrophysiology and muscle biopsy. A neurologist specialized in muscle disease (MP) scored whether the thus established phenotype was compatible with any of the described distal myopathies using the following ratings:

- Typical: if the clinical features were similar to those described in the literature
- Consistent but not strictly typical: if the clinical features were quite similar to the ones described in the literature but had additional or missing features
- Different: neither of the above

The distal myopathies included as differential diagnoses were the 15 subtypes with a known genetic basis reported in a previous classification (6) and four conditions that can present with distal weakness but are not classified as distal myopathies: distal nebulin myopathy with nemaline bodies (8), *DNM2*-associated centronuclear myopathy, hereditary myopathy with early respiratory failure (HMERF), inclusion body myositis (IBM).

### Developing a literature based distal myopathies MRI template

A literature search was performed to identify all publications describing CT/MRI muscle involvement in genetically confirmed distal myopathies published up to January, 1st 2015. Studies were identified on Pubmed by manual search including related citations and key author searches. Search terms used were [MRI] or [CT] and each of the 15 distal myopathies with a known genetic basis (6) and the four additional conditions listed above. Single case reports and case series with <5 patients were excluded except for diseases in which this was the only information available. Only studies with genetically confirmed patients were considered. In *ANO5* and *DYSF* associated muscle diseases that may have phenotypes other than distal myopathy, only articles focusing on distal myopathy phenotypes were included. The two reported phenotypes with distal involvement for *TTN* mutations, tibial muscular dystrophy (TMD) (9) and hereditary myopathy with early respiratory failure (HMERF) (10) were grouped separately. As T1-weighted sequences were the one sequence most frequently used in all MRI studies, only publications using this sequence were considered. For each study we assessed the number of patients imaged, the rating scale applied, if any, and the availability of individual muscle scores for each patient.

For each genotype we summarized the most important imaging features in a schematic drawing and in a table. Thigh and calf muscles and where possible, early and late disease stages, were represented separately. The schematic drawings were then summarized into one template.

The template was tested to ensure that it reflects the description in the literature. Seven random images were chosen from the evaluated manuscripts and were scored by three examiners, two neuroradiologists and one neurologist (TAY, SS, JMM) by consensus. Each image was scored against each possible gene using a previously used classification (5):

- Typical: if they were similar to the patterns reported in the literature
- Consistent but not strictly typical: if the patterns observed were quite similar to those observed but had additional or missing features
- Different: neither of the above

### Diagnostic utility of MRI

We assessed the diagnostic utility of the thus developed MRI template in terms of its sensitivity and specificity and potential impact on the diagnostic workup of the patients audited.

#### MRI pattern analysis

Three examiners blind to all genetic and clinical data compared each scan with the literature based MRI template using the scoring system described above. All MRI scans were included in the analysis including normal MRI. In cases of uncertainties, the original publication was reviewed. Patterns were agreed on by consensus, combining the information from thigh and calf muscles. The reasons for pattern allocation were recorded by an observer not involved in the evaluation. Based on the pattern analysis a list of MRI dependent diagnoses was created for each patient. In case of existing genetic data, the test results were reviewed to assess if the diagnosis was concordant and if the genes suggested by MRI were tested. If the genetic data was absent, genetic analysis was performed through single gene testing or screening of all listed distal myopathy genes. The latter was done using data obtained from Agilent Sure Select Focused Exome (Agilent, California, USA) according to the manufacturer's protocol. We limited the analysis of the *TTN* variants to the ones associated with the diseases under study. Details on sequencing protocols and bioinformatics pipeline are available by request.

#### Sensitivity and specificity of the literature based template

The sensitivity and specificity of the MRI pattern derived diagnosis was determined when a genetic diagnosis was available. All MRIs including normal and uninformative scan were included in the assessment.The reasons for discrepancies between the MRI and the genetic diagnosis were recorded. Typical and consistent classifications were labeled as positive diagnosis. Sensitivity was calculated as true positives/(true positives + false negatives) and specificity as true negatives/(true negatives + false positives).

#### Contribution of MR template to diagnostic work-up

To evaluate the added value of MRI pattern analysis in the diagnostic work-up we compared the list of candidate genes generated by clinical re-evaluation (under Diagnostic Re-Evaluation) with those suggested by the MRI pattern analysis (MRI Pattern Analysis). The comparison between groups was made using the Mann-Whitney *U*-test.

## Results

### Patient selection

We identified 55 patients (33 male, 22 female) who met the inclusion criteria. In 22 of them a genetic cause had been identified (Table 1).

**Table 1:** Genetic variants identified in our cohort.

**ID**	**Gene**	**Variant**	**Predicted protein effect**
1	DYSF	NM_003494:c.3805dupG	p.E1269Gfs^*^7
		NM_003494:c.5698_5699delAG	p.S1900Qfs^*^14
3	DYSF	NM_003494:c.3051dupC	p.I1018Hfs^*^13
		NM_003494:c.5803_5811dupCCAGCCAAG	p.P1935_K1937dup
4	MYOT	NM_006790:c.179C>G	p.S60C
5^*^	MYH7	MYH7:NM_000257:c.5537G>A	p.R1846H
10	MYH7	NM_000257: c.4317_4319del	p.A1439del
11	GNE	NM_001128227:c.796_797insCCAAT	p.L266Sfs^*^3
		NM_001128227:c.2179G>A	p.V727M
18^*^	TTN	NM_001267550:c.95187G>C	p.W31729C
21^*^	GNE	NM_001128227:c.1225G>T	p.D409Y
		NM_001128227: c.922C>T	p.R308C
22	GNE	NM_001128227:c.1646G>A	p.G549D
		NM_001128227:c.2179G>A	p.V727M
28^*^	MYOT	NM_006790:c.179C>G	p.S60C
29	DYSF	NM_003494:c.2858dupT	p.F954Vfs^*^2
		NM_003494:c.526C>T	p.Q176^*^
30	DYSF	NM_003494:c.4200dupC (hom)	p.I1401Hfs^*^8
31	DES	NM_001927:c.46C>T (hom)	p.R16C
32	MYOT	NM_006790:c.179C>G	p.S60C
35	MYOT	NM_006790:c.179C>G	p.S60C
40	DYSF	NM_003494:c.4200dupC (hom)	p.I1401Hfs^*^8
41	MYH7	NM_000257:c.4522_4524del	p. E1508del
44	MYOT	NM_006790:c.179C>G	p.S60C
49	MYH7	NM_000257:c.4522_4524del	p. E1508del
50	VCP	NM_007126:c.277 C>T	p.R93C
54	GNE	NM_001128227:c.740T>C	p.V247A
		NM_001128227:c.1985C>T	p.A662V
60	TTN	NM_001267550:c.95134T>C	p.C31712R

* *Variants identified during the study*.

The cohort included 10 patients with childhood disease onset (<10 years old), 22 patients with juvenile/adult onset (10–39 years) and 23 patients with late onset (>40 years old) disease. The median age was 56 years (range 24–84 years).

### Assessment of diagnostic workup

#### Audited cohort and data collection

The diagnostic work up was audited in 38 patients in whom all required data were available (Table 2). At time of data collection a genetic diagnosis had been established in 15 patients (39%) but in only 5/17 (29%) in the late onset patient group. The median time from the onset of symptoms to establishing a genetic diagnosis was 12.1 years (range 1.7–40 years, *n* = 15). Six patients were genetically confirmed prior to their first evaluation. Seven (18%) patients have received a previous diagnosis of a neuropathy and five (13%) a diagnosis of IBM before they were reclassified as distal myopathy. Three of these now have a confirmed genetic diagnosis (*MYH7* and *TTN* genes in the neuropathy group and *MYOT* in the IBM group).

**Table 2:** Main clinical features of the audited distal myopathy cohort.

**Group**	**Onset years median (range)**	**% patients with a genetic diagnosis (*n*)**	**Genes identified (*n*)**	**Muscle involvement other than distal weakness (*n*)**	**Ambulation (*n*)**	**Predominant muscle pathology (*N*/total patients biopsied)**
Childhood (0–10 years) *n* = 6	4 (0–8)	50% (3)	MYH7 (3)	Proximal weakness (2)	Independent (4) walking aids (1) wheelchair (1)	Fibre type disproportion (1/4), ring binden (1/4), core fiber (1/4), mild myopathic (1/4)
Juvenile/adult (10–39) *n* = 15	22 (16–37)	46.7% (7)	DYF (5) DES (1) GNE (1)	Proximal weakness (5) Ptosis (2) Laryngeal involvement (2)	Independent (14) wheelchair (1)	Rimmed vacuoles (3/14), nemaline bodies (2/14), myofibrillar myopathy (2/14), myopathic with pathological immunostaining for dysferlin (4/14), vacuoles with fibrillar material (1/14), angular fibers (1/14), no abnormalities (1/14)
Late onset (≥40) *n* = 17	53 (40–68)	29.4% (5)	MYOT (4) GNE (1)	Proximal weakness (6)	Independent (9) walking aids (5) wheelchair (3)	Rimmed vacuoles (3/16), myofibrillar myopathy (6/16), core fiber (2/16), mild myopathic (2/16), dystrophic process (1/16), no abnormalities (1/16), end stage myopathic (1/16)

*The patients are grouped based on age of onset (1)*.

Four patients had a neuropathic EMG with a muscle biopsy showing a myopathic process. In a fifth patient the biopsy showed angular fibers more consistent with a chronic spinal muscular atrophy while the EMG was myopathic with fibrillations. Rimmed vacuoles were the most frequent pathological feature (13/38, 34%) especially in the late onset group.

The review of the diagnostic workup showed that EMG and muscle biopsy were performed in almost all patients (34/38) whereas muscle MRI was performed in 24 patients. Eleven out of 38 patients (29%) had a second muscle biopsy and in four of them the second biopsy provided additional information (i.e., presence of rimmed vacuoles). Electrophysiological investigations were performed first and within 1 year of the initial appointment. There was no established order for biopsy and MRI, and 58% (11/19) of patients had their muscle MRI within 1 year. In four patients the MRI was done before the second biopsy and in two patients the second MRI guided biopsy was more informative than the first biopsy (e.g., rimmed vacuoles). In all 38 patients, 151 DNA tests of genes causing distal myopathy were performed, but in only 15 were found pathogenic variants (10%).

#### Diagnostic re-evaluation

**Figure 1:**
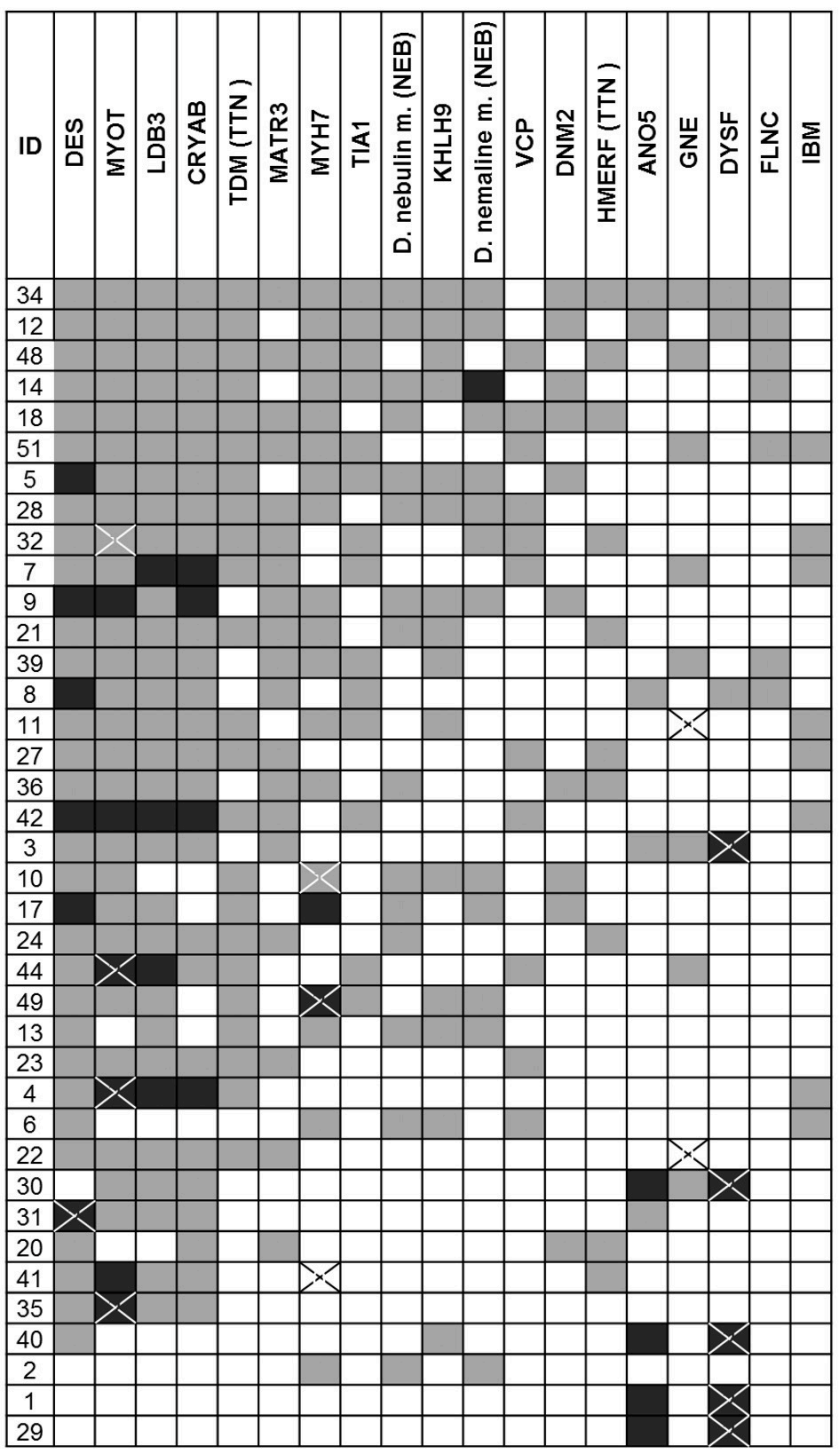
Results of the clinical-pathological re-evaluation. The diseases are reported using gene symbols apart for IBM. For *TTN* and *NEB* two different phenotypes were considered. *FLNC* refers to the phenotype described in the distal ABD-filaminopathy. In black the disease scored as typical and in gray as consistent. A cross indicates the gene confirmed as positive. HMERF, hereditary myopathy with early respiratory failure; TMD, tibial muscular dystrophy.

Based on the clinical evaluation, the number of genes per patient that were scored as typical or consistent with the published clinical phenotype was high (median 8, range 2–17, total 315) but only 10% of these (32/315) were categorized as typical (Figure 1). Mutations in the *DES, MYOT* and *LDB3* genes were considered possible causes in the majority of patients (respectively in 34/38, 31/38, 31/38; >80% of patients), whilst *DYSF* and *ANO5* related muscle diseases were suggested less frequently (8/38 and 9/38, <25% of patients), but when suggested had a clinical phenotype more commonly categorized as typical (5/8 and 4/9 respectively).

In 15 patients with a genetic diagnosis (*n* = 15) the number of genes suggested by clinical re-evaluation per patient was still high (median 6). In 12 patients the disease causing gene was among those suggested but not in the remaining three patients (two with *GNE* mutations and one with an *MYH7* mutation). In 8/38 (21%) patients a diagnosis of IBM was suggested.

### Developing a literature based distal myopathies MRI template

Twenty-one publications on muscle MRI patterns in distal myopathies that met the inclusion criteria were identified, (8, 10–29) and are summarized in Supplementary Table 1. The number of patients available per-publication is small and varies widely (median 8, range 1–32). For all diseases only one or two articles were available, except *MYOT*-associated myopathy where there were three.

Twelve articles (57%) applied a semi-quantitative scoring system but only eight of these reported the individual muscle scores for each patient. The most common rating scale applied was the modified 5-point scale (14). In the other nine articles the patterns were only described in the discussion section, making the integration, interpretation, and comparison of data difficult. For seven diseases the pattern was based on descriptive data only.

**Figure 2:**
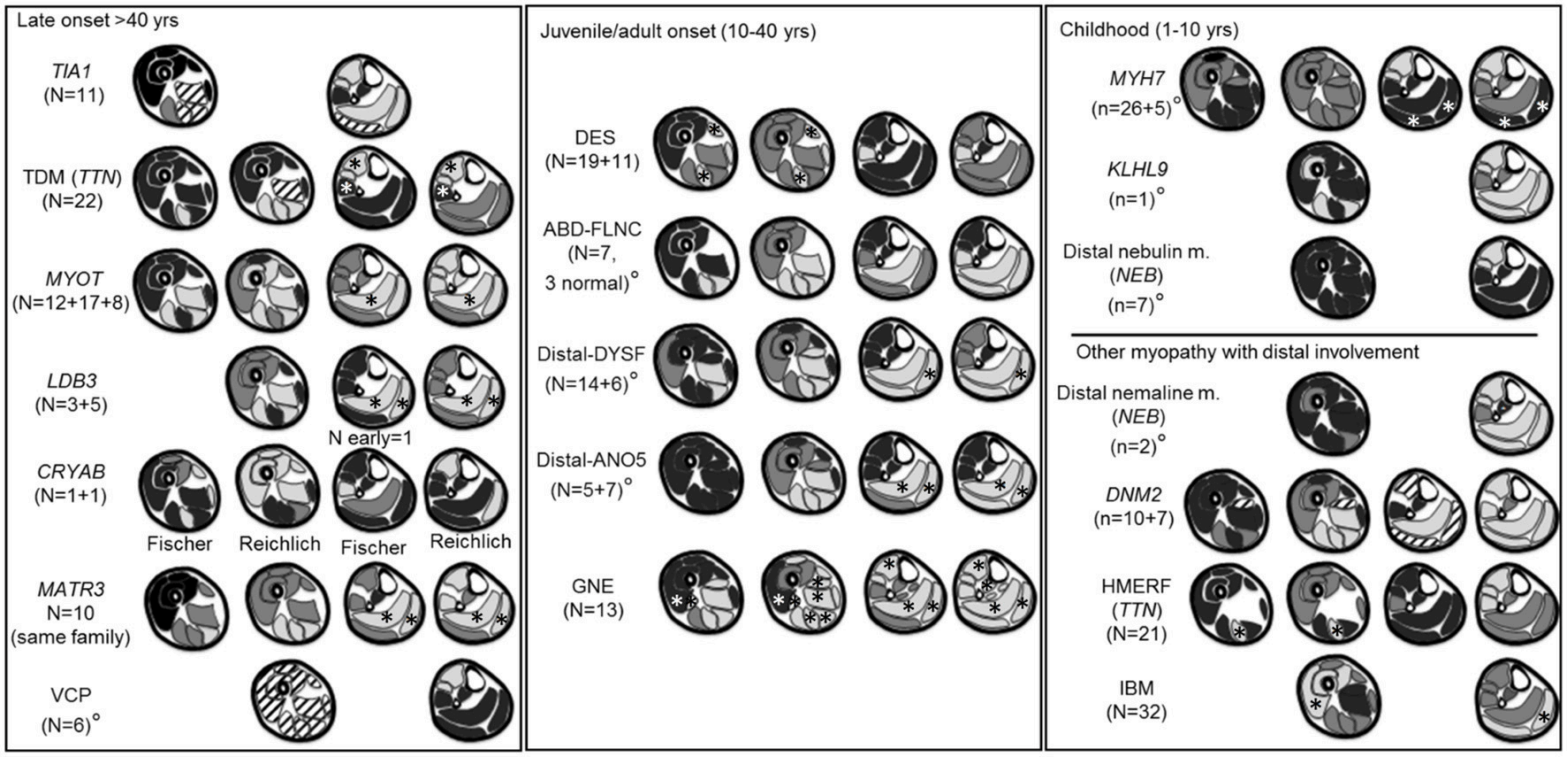
Literature based patterns. For every disease we reported name, gene and number of patients per article. A gray scale matching appearances on T1-weighted images was used to indicate the degree of involvement. Black indicated a muscle generally spared, dark gray a muscle less severely or less frequently involved, light gray a muscle most severely or most frequently involved. If muscles were not reported in the literature, such as the adductor longus, they were omitted from the pattern diagram. From left to right are represented early and late involvements. °Near the disease's name indicates pattern for whom the individual muscle score was not available. ^*^Muscles reported as either almost never or almost always involved. Stripes: discordant or highly variable involvement; indicating limited use for pattern assessment.

A diagram of the MRI pattern of each genetic disease is shown in Figure 2; the detailed description is provided in the Supplementary Table 1. Each pattern represents an integration of the information provided in the available publications. In 7/19 diseases, only an overall pattern without a temporal distinction could be compiled. For the *CRYAB* gene, two publications reported on one patient each describing completely different patterns. In this instance, both patterns were included in the summary.

The evaluation of seven images from the above mentioned publications (10, 12, 16, 20, 22, 23) using the MRI template resulted in a median of 2 (range 2–5) genes per evaluated scan graded typical or consistent. The correct gene was suggested in every case (typical *n* = 5, consistent *n* = 2).

### Diagnostic utility of MRI

#### MRI pattern analysis

**Figure 3:**
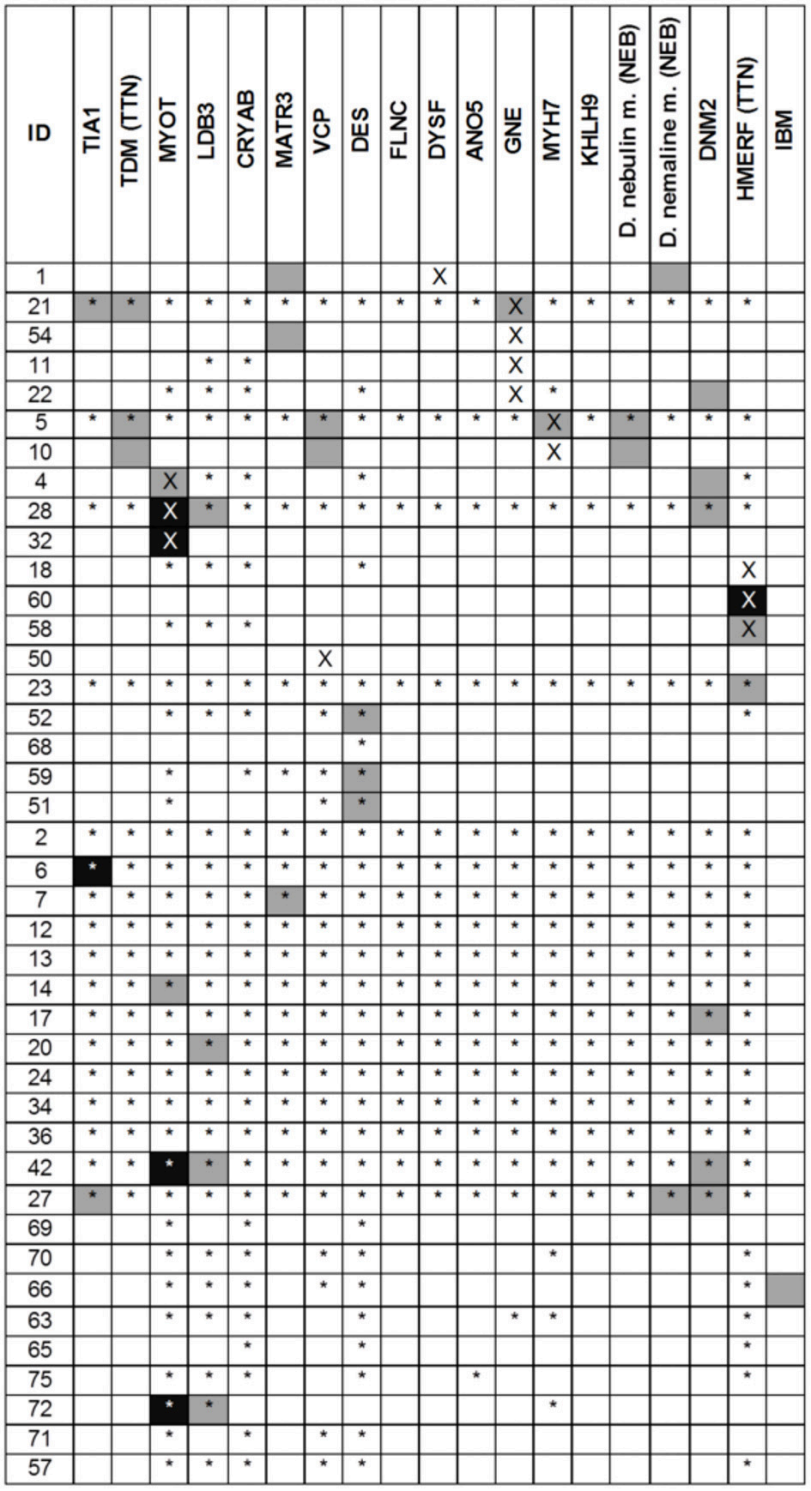
Results of the MRI pattern analysis. In black the disease scored as typical and in gray as consistent. A cross indicates the gene identified as pathogenic whereas the star indicated a negative genetic test. For *TTN* and *NEB* two different phenotypes were considered. Variants of uncertain significance in *DES* gene were found in ID 23, 52, 68, 59, 51.

Muscle MRI patterns were assessed in 41 patients in whom MRI examinations were available of which 14 had a genetic diagnosis. The number of genes suggested by the muscle MRI patterns was low (median 1, range 0–4) (Figure 3). The most commonly suggested genes were *MYOT* and *DNM2*; their MRIs were scored as typical or consistent in 6/41 patients. Eighteen (44%) patients had a pattern that was considered to be not typical or consistent with any published pattern. Of these 18 patients, one had a normal MRI, three had severe and diffuse involvement of all muscles and the remaining 14 patients (34%) had a different pattern from any published. In 7 of the 14 patients with a genetic diagnosis the disease causing gene was among those suggested by MRI.

In five patients *DES* variants of unknown significance (VUS) were found: c.1243C>T, p.R415W (*n* = 2), c.1372-15 T>A (*n* = 2, related), c.49_54dupACCTTC (*n* = 1). The latter also carried a variant in *MYOT* (c.220C>A). In three cases the pattern analysis supported a *DES*-related myopathy.

#### Sensitivity and specificity of the literature based template

In the patients with a genetic diagnosis (*n* = 14), muscle MRI pattern analysis suggested the causative gene in seven (50%) patients and an incorrect genetic diagnosis in the remaining seven patients. The reasons for this mismatch are as follows:

1. *MYH7* (*n* = 1): Involvement of the gastrocnemius medialis muscle which is not mentioned in the pattern description.
2. *TTN* (*n* = 1): Mild thigh involvement, and consistent with the pattern, but severe calf involvement without a characteristic pattern.
3. *GNE* (*n* = 3): (a) Sparing of the rectus femoris muscles and involvement of the vastus lateralis muscles at an advanced stage (*n* = 1); (b) severe involvement of the gastrocnemius lateralis muscle, which was more affected than the gastrocnemius medialis muscles (*n* = 1); (c) sparing of the short head of the biceps femoris muscle, which is typically affected in *GNE*-myopathy (*n* = 1).
4. *VCP* (*n* = 1): Involvement of the posterior calf, whereas according to the pattern the involvement is anteriorly.
5. Miyoshi myopathy (*DYSF*) (*n* = 1): Sparing of the adductor magnus muscle. No calf images were available.

The overall sensitivity and specificity of the template based pattern MRI pattern was 50% and 32% respectively. The sensitivity for single diseases (*MYOT, MYH7, GNE, TTN*-HMERF) was quite variable (0–100%) but the specificity was high (90–100%) (Table 3).

**Table 3:** MRI pattern sensitivity/specificity in genetically confirmed patients.

	**Prevalence**	**Sensitivity**	**Specificity**	**PPV**	**NPV**
Total^*^	14/41 (34)	7/14 (50) [23–77]	6/19 (32)^∧^ [13–57]	7/20 (35) [23–50]	6/13 (46)^∧^ [27–67]
MYOT	3/41 (7)	3/3 (100) [29–100]	27/30 (90) [73–98]	3/6 (50) [25–74]	27/27 (100) –
GNE	4/41 (10)	1/4 (25) [1–81]	17/17 (100) [80–100]	1/1 (100) –	17/20 (85) [76–91]
MYH7	2/41 (5)	1/2 (50) [1–99]	20/20 (95) [83–100]	1/1 (100) –	20/21 (95) [83–99]
TTN-HMERF	3/41 (7)	2/3 (67) [9–99]	24/25 (96) [77–100]	2/3 (66) [20–94]	24/25 (96) [83–99]
VCP	1/41 (2)	0/1 (0) [0–97]	23/25 (92) [74–99]	0/2 (0) –	23/24 (96) [95–96]
DYSF	1/41 (2)	0/1 (0) [0–97]	17/17 (100) [80–100]	0/0 (0) –	17/18 (94) [94]

*Values are n (%) and 95% confidence interval are reported in square bracket. In 9/41 patients >1 pattern suggested from 19 different choices available (median 1, range 0–4). Prevalence, number of patients with the disease divided by all evaluated patients; True positive, patients with an MRI pattern typical/consistent for the diagnosis and confirmed by genetic testing; False positive, patients with an MRI pattern typical/consistent for the diagnosis, proven incorrect by genetic testing; True negative, patients with an MRI pattern that differs from the described MRI pattern; genetic testing confirms that the diagnosis is different; False negative: Patients with an MRI pattern that differs from the described MRI pattern; genetic testing confirms the diagnosis*.

* *When assessing the overall specificity and sensitivity, we considered: True positive (n = 7): All patients with at least one MRI pattern typical/consistent for the diagnosis; confirmed by genetic testing. False positive (n = 13): All patients with at least one MRI pattern typical/consistent for the diagnosis, proven incorrect by genetic testing*.

∧ *True negative (n = 6): All patients with an MRI pattern that differs from the typical MR pattern; testing of all 16 causative genes confirms the absence of a genetic diagnosis. Eight patients in whom only a fraction of genes were screened were therefore excluded. False negative (n = 7): All patients with an MRI pattern that differs from the described MR pattern; genetic testing confirms a diagnosis. Sensitivity, true positives/true positive + false negative. Specificity, true negative/true negative + false positive; Positive predictive value (PPV), true positive/true positive + false positive; Negative predictive value (NPV), true negative/true negative + false negative*.

In the five patients with *DES* VUS, the MRI pattern analysis suggested *DES* as a causative gene in three of them. In the remaining two the pattern was considered different as reported below:

Patient ID 23 (c.1243C>T, p.R415W): The thigh involvement was mild, and consistent with the pattern including marked semitendinosus involvement. However, the calf was severely involved without a characteristic pattern.

Patient ID 68 (c.1372-15 T>A): Widespread muscle involvement with no recognizable pattern.

#### Contribution of the MRI template to the diagnostic work-up

In 24 patients of the audited cohort (*n* = 38) who underwent muscle MRI, we compared the diagnosis suggested by MRI with the ones obtained by clinical re-evaluation.

The number of genes suggested by MRI pattern analysis was significantly smaller compared to clinical re-evaluation (MRI, *n* = 31, median 1 (0–4); clinic, *n* = 210, median 9 (2–17), *p* < 0.0001; Table 4). However the number of genes correctly predicted was higher by clinical re-evaluation than by MRI pattern analysis (7/10 vs. 5/10). MRI pattern analysis did not suggest IBM as likely diagnosis in any case whereas clinical re-evaluation considered IBM consistent in 7/24 (29%) of patients.

**Table 4:** Comparison between clinical re-evaluation and MRI pattern analysis.

**ID**	**Clinical re-evaluation**	**MRI pattern analysis**	**Gene**
1	**j**,k	f,p	**j**
2	m,o,p	–	–
4	b,**c**,d,e,h,s	**c**,q	**c**
5	a,b,c,d,e,h,**m**,n,o,p,q	b,g,**m**,o	**m**
6	a,g,h,m,n,o,s	a	–
7	a,b,c,d,e,f,g,h,l,s	f	–
10	b,c,h,**m**,n,o,p,q	b,g,o	**m**
11	a,b,c,d,e,h,m,n,s	–	**l**
12	a,b,c,d,e,h,i,j,k,m,n,o,p,q	–	–
13	b,e,h,m,n,o,p	–	–
14	a,b,c,d,e,h,I,m,n,o,p,q	c	–
17	b,c,d,h,m,op,q	q	–
18	b,c,d,e,f,g,h,m,o,p,q,**r**	–	**r**
20	e,f,h,q,r	d	–
21	b,c,d,e,f,h,m,n,o,r	a,b,**l**	**l**
22	b,c,d,e,f,h	q	**l**
23	b,c,d,e,f,g,h	r	–
24	b,c,d,e,f,h,o,r	–	–
27	b,c,d,e,f,g,h,r,s	a,p,q	–
28	b,**c**,d,e,f,g,h,m,n,o,p	**c**,e,q	**c**
32	a,b,**c**,d,e,f,g,h,p,r,s	**c**	**c**
34	a,b,c,d,e,f,h,I,j,k,l,m,n,o,p,q,r	–	–
36	c,d,e,f,h,m,o,q,r	–	–
42	a,b,c,d,e,f,g,h,s	c,d,q	–

*For the comparison we combine both typical and consistent score as positive. Clinical re-evaluation and MRI analysis were performed as reported in methods Diagnostic Re-Evaluation and MRI Pattern Analysis. Gene indicates the gene identified in the patient and it is highlighted in bold. a, TIA1; b, TDM (TTN); c, MYOT; d, LDB3; e, CRYAB; f, MATR3; g, VCP; h, DES; i, ABD-FLNC; j, DYSF; k, ANO5; l, GNE; m, MYH7; n, KLHL9; o, Distal nebulin myopathy (NEB); p, distal nebuline myopathy with nemaline bodies (NEB); q, DNM2; r, HMERF (TTN); s, IBM*.

## Discussion

To assess the potential role of MRI in improving the diagnostic work-up, we established a literature based template of MRI patterns in distal myopathies. When applied in a clinical setting, MRI pattern analysis was able to correctly identify diseases such as *MYOT*-related myopathy and rule out mimicking diseases as IBM. This study is the first to assess the utility of muscle MRI pattern in a non-selected cohort of distal myopathy patients.

The diagnostic work-up of distal myopathies can be long and complex. The median time from onset of symptoms to diagnosis was 12.1 years with an initial genetic diagnostic rate of 39% and a misdiagnosis rate of 29%. We identified three potential causes of delay

The first was the high number of non-specific muscle biopsies highlighted by the frequency of repeated biopsies (29%). In 4/11 (36%) patients the second biopsy was more informative indicating a possible role of MRI in optimizing the muscle selection.

The high number of genetic tests requested (*n* = 151 considering only distal myopathy genes) was the second cause for delay. This high number was due to overlapping clinical features within the group of distal myopathies as well as the uncertainty about which gene to sequence first. The current use of next generation sequencing in clinical practice will most likely reduce this delay, but an additional problem of finding multiple genetic VUS per patient will be introduced.

The third important cause for diagnostic delay was the difficulty in distinguishing a distal myopathy from a neuropathy or IBM. 7 of the 38 (18%) patients audited were initially diagnosed with a neuropathy and 5 (13%) patients with IBM before being re-classified as a distal myopathy. Motor neuropathies and distal myopathies are difficult to differentiate clinically because of the shared distal weakness, the presence of denervation signs in distal myopathies and the possible association of the two disorders (30–33). With IBM, distal myopathies may share late onset weakness and the presence of rimmed vacuoles in biopsies.

It has been suggested that MRI pattern analysis can support the diagnostic process. The review of the current available literature on muscle MRI patterns in distal myopathy revealed the heterogeneity of these patterns which were thought to be characteristic for each gene. In seven diseases the patterns were based on small case series, the majority of muscle MRI patterns (10/19) were described in one publication and in 57% (12/21) of publications the individual muscle scores for each patient were not available.

Our literature review showed that some muscle MRI patterns which were reported to be specific in certain diseases were similar to those of other diseases. This is the case in *DES* associated myopathy and HMERF where an early involvement of the semitendinosus and peroneal muscles has been described (10, 12, 14). Some of these problems could be addressed by focusing on the identification of key MRI features rather than the overall pattern of muscle involvement such as the biceps femoris short head involvement in GNE-myopathy (22). All publications except one reported a thigh involvement at some stage of the disease, suggesting that assessing both the thigh and calf pattern would be advantageous, especially in advanced stages of a disease when the calf muscles could be completely replaced by fatty tissue.

To assess the potential diagnostic role of a muscle MRI pattern analysis it is important to determine its sensitivity and specificity and assess its value in the clinical work up. However, determining sensitivity and specificity of a diagnostic test in rare diseases is challenging as inevitably, large confidence intervals limit the validity of the test. It is therefore not surprising, that the sensitivity of our MRI template in identifying the correct genetic disease was highly variable, and overall quite modest. Specificity was low considering overall pattern analysis, however, was high for some diseases, such as *MYOT, MYH7, GNE*-related myopathy, and *TTN*-HMERF However the low prevalence of these diseases and the limited number of cases may have overestimated these values. Wider cohort of genetically confirmed patients are needed for accurately determine sensitivity and specificity.

**Figure 4:**
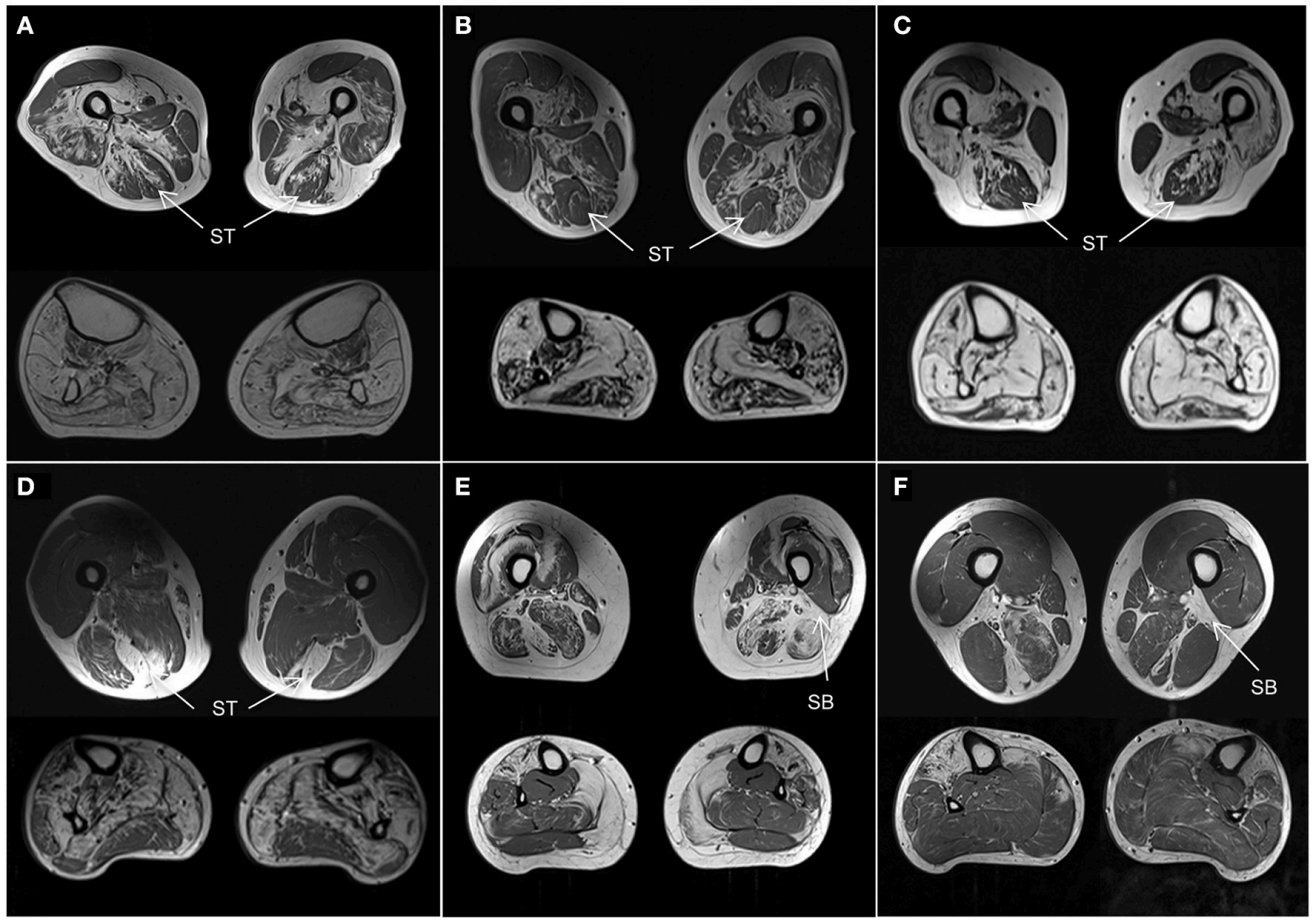
Muscle MRI patterns. Axial T1-weighted images at mid-thigh and mid-calf level in selected subjects. **(A–C)** Patients with genetically confirmed *MYOT* mutations all correctly identified. They are all characterized by a relative sparing of the semitendinosus (ST) muscle. The vastus intermedius and sartorius muscles are more affected than the gracilis muscle. All calf muscles can be affected; the gastrocnemius lateralis muscle is usually less affected than the medialis. **(D)** Patient with *TTN* (ID18) mutation. He was not correctly identified as the calf was severely involved without the characteristic pattern. However the selective involvement of semitendinosus (ST) within the thigh is characteristic and could represent a key feature of this disease. **(E,F)** Patients with *GNE* mutations. One patient (**E**, ID11) was not correctly identified. The rectus femoris muscles were spared and the vastus lateralis muscles were involved contrary to the reported pattern. However both patients **(E,F)** revealed severe involvement of short head of biceps femoris (SB) muscles which could represent a key feature of the disease.

Although the number of individuals with the same genetic disease was small, some observations of the muscle MRI pattern of these diseases can be made. Using our template we correctly identified all three *MYOT*-associated myopathies, which is also one of the best described in the literature (Figures 4A–C). Also 2 of 3 patients with *TTN*-HMERF were correctly identified. In third patient the MRI pattern did not suggest the correct diagnosis. However, the patient showed an early involvement of the semitendinosus muscle, suggesting that this could be an easy recognizable key feature, which could improve the identification rate. This also applies to *GNE*-myopathy, where the MRI pattern predicted the correct diagnosis in only one of 4 patients. A prominent involvement of the short head of the biceps femoris muscle was noted in 3 patients, suggesting that this could also serve as a key feature, as reported (Figure 4) (22).

A comparison of MRI pattern analysis with clinical re-evaluation (Contribution of the MRI Template to the Diagnostic Work-Up) showed that MRI suggested a lower number of genes (per patient median 1 instead of 8 respectively). In the audited cohort, this would have therefore shortened the diagnostic process in 5/10 genetically confirmed diseases. Conversely, in 2 patients MRI pattern analysis excluded the correct diagnosis which was included in the larger number of genes suggested by clinical evaluation. In 3 patients neither method identified the correct gene. Considering that, care should be given to not exclude diseases only on the basis of MRI pattern analysis. In the current NGS era the utility of MRI in addressing single genetic testing is limited. However, MRI pattern analysis suggesting a limited number of candidate genes per patient may be helpful in supporting NGS analysis when VUS are detected. In our cohort for example, 3 out of 5 patients carrying *DES* VUS have an MRI pattern suggestive of *DES*-related myopathy. Given the current limited data available on MRI pattern we cannot draw a conclusion on the pathogenicity of these variants, but it represents an example of the use of MRI in the diagnostic process of inherited myopathies.

Our analysis has also shown that MRI helps reducing the frequency of misdiagnosis. Indeed, misclassification as IBM was one of the major causes of delays in our audited cohort. According to our template however, the muscle MRI pattern in these patients was not consistent with a diagnosis of IBM (29, 34). MRI could therefore raise the suspicion of a genetic distal myopathy in a patient misdiagnosed as IBM and therefore prompt the required genetic investigations.

In conclusion, our study showed that the application of muscle MRI can be useful for targeting the best muscle to biopsy, guide the genetic testing, interpret complex genetic results obtained by NGS and help avoiding misdiagnosis (IBM).

Currently, the low overall sensitivity and specificity makes it difficult to advocate the general use of muscle MRI in the diagnostic work-up of distal myopathies, especially in a resource limited environment. However, MRI pattern analysis could play a central role in the context of next generation sequencing where a considerable quantity of sequencing data are generated and the resulting difficulties in distinguish pathogenic variants from rare but benign polymorphisms. In this scenario, MRI pattern analysis could help in guiding the selection of the appropriate variant, which would help to avoid muscle biopsies, thereby decreasing the invasiveness and in turn the costs of the diagnostic pathway. The next step is therefore to assess the value of MRI in determining the pathogenic variant in a prospective cohort undergoing broad next generation sequencing screening.

## Author contributions

All authors edited and approved the final version of the manuscript. EB, JM, MR, HH, MP, MH, VS, and TY contributed to study design. EB, JM, SS, CW, DL, AP, EM, MP, and TY contributed to data acquisition and analysis. EB, JM, TY, and VS contributed to manuscript drafting. MR, HH, MH, SS, CW, DL, AP, EM, and MP contributed to critical revision of the manuscript.

### Conflict of interest statement

The authors declare that the research was conducted in the absence of any commercial or financial relationships that could be construed as a potential conflict of interest.
