# Supplementary material for: The Diagnostic Value of MRI Pattern Recognition in Distal Myopathies
Source: Front Neurol. 2018 Jun 26;9:456. doi: 10.3389/fneur.2018.00456 (PMC6028608; doi:10.3389/fneur.2018.00456)
Supplement: Supplementary file 1 [file Table_1.DOCX]

Supplementary Material

The Diagnostic Value of MRI Pattern Recognition in Distal Myopathies

**Enrico Bugiardini, Jasper M. Morrow, Sachit Shah, Claire L. Wood, David S. Lynch, Alan M. Pitmann, Mary M. Reilly, Henry Houlden, Emma Matthews, Matt Parton, Michael G. Hanna, Volker Straub, Tarek A.Yousry***

*** Correspondence:** Professor Tarek A. Yousry [t.yousry@ucl.ac.uk](mailto:t.yousry@ucl.ac.uk)

# Supplementary Table 1: literature review and patterns summary.

| **Disease** | **Reference** | **Total no. of**  **scans** | **Age**  **Range**  **(at MRI)** | **Scoring system applied** | **Raw data available** | **Control group (C)/**  **other disease compared (D)** | **Overall pattern summary thigh** | **Overall pattern**  **summary leg** |
| --- | --- | --- | --- | --- | --- | --- | --- | --- |
|  |  |  |  |  |  |  |  |  |
| Welander  distal  myopathy  (WDM)  (*TIA*) | Mahjneh *et al*. 2004 | 11 (MRI) | 45-86 | 4 grade scale  M0: Normal  M1: patchy but not confluent intramuscular signal.  M2: patchy and confluent intramuscular signal, involving <50% of individual muscle volume.  M3: Homogeneously hyperintense; confluent intramuscular signal involving over 50% of individual muscle volume | Y | D: WDM vs TMD | Reported some patients with proximal involvement in the posterior compartment without a specific pattern. | Involvement of TA, EDL, Sol and GM. Variable involvement GL.  Sparing TP and peroneus group (apart isolated cases). |
|  |  |  |  |  |  |  |  |  |
| Tibial  Muscular  Dystrophy  (TMD)  (*TTN*) | Mahjneh *et al*. 2004 | 22 (MRI) | 48–85 | Same as above | Y | D: WDM vs TMD | Proximal involvement in 50% patient.  The first muscle affected is ST. In advanced stage involvement of SM and BF.  Spared: VL, RF. | Early involvement of TA and EDL. Later focal changes may occur in soleus and gastrocnemius.  Spared: peroneus. |
|  |  |  |  |  |  |  |  |  |
| *MYOT*-myopathy | Olive *et al.* 2011 | 17 (MRI or CT) | NA | Modified 5-point scale (Fisher *et al.* 2008) | N | D: *DES* vs *MYOT* vs *LDB3* vs genetically unconfirmed MFM | Early involvement of SM, BF, AM. Later VI,VM.VL less affected.  ST reported as mild affected or spared.  Relatively spared RF, S, G.  Sartorius was at least equally affected as the semitendinosus (S≥ST) | The first muscles affected are Sol and GM with relatively sparing of GL. Subsequently the TA and P are involved.  Reported involvement of TP. |
|  | McNeill *et al.* 2009 | 8 (MRI) | 53-76 | 4 grade scale  0=normal  + (mild): <30% of the muscle volume affected,  ++ (moderate): as 30–60% affected  +++ (severe involvement):> 60% involved | Y | None |  |  |
|  | Fischer *et al.* 2008 | 3 MRI+9 CT | 43-85 | Modified 5-point scale  0: normal  1 (mild) traces of increased signal intensity  2 (moderate): increased signal intensity with beginning confluence in less than 50% of the muscle;  3 (severe): increased signal intensity in more than 50% of an examined muscle  4 (end-stage disease) the entire muscle is replaced by increased signal intensity | Y | D: *DES* vs *MYOT* vs *LDB3* vs *FLN*C vs *CRYAB* |  |  |
|  |  |  |  |  |  |  |  |  |
| *LDB3-*myopathy | Olive *et al*. 2011 | 5 (MRI or CT) | NA | Modified 5-point scale (Fisher *et al*. 2008) | N | D: DES vs MYOT vs LDB3 vs genetically unconfirmed MFM | Involvement of SM, BF, AM.  Vastus affected but less degree.  ST spared. | Early involvement of GM and Sol. In advance stage all the calf could be severely affected. |
|  | Fischer *et al.* 2008 | 3 (MRI)* | 54-66 | Modified 5-point scale (Fisher *et al.* 2008) | Y | D: DES vs MYOT vs LDB3 vs FLNC vs CRYAB |  |  |
|  |  |  |  |  |  |  |  |  |
| *CRYAB-*myopathy | Fischer *et al.* 2008 | 1 (CT) | NA | Modified 5-point scale (Fisher *et al.* 2008) | Y | D: DES vs MYOT vs LDB3 vs FLNC vs CRYAB | Different pattern reported  Fisher *et al.* 2008 Involved ST,S,G  Reilich *et al*. 2010  Involved VI,VM,VLRF,AM,ST. | Different pattern reported  Fisher *et al.* 2008  Involved P  Reilich *et al*. 2010  Involved GM, TA. |
|  | Reilich *et al.* 2010 | 1 (MRI) | NA | None | N | None |  |  |
|  |  |  |  |  |  |  |  |  |
| *MATR3*-myopathy | Muller *et al.* 2014 | 10 (MRI) | 54-66 | Modified 5-point scale (Fisher *et al*. 2008) | Y | None | Posterior compartment, especially the SM was more severely affected, and the quadriceps muscle was relatively spared. | Early and severe involvement Sol and GM.  The extent of fatty replacement was: Sol>GM>TA>GL>>TP |
|  |  |  |  |  |  |  |  |  |
| *VCP*-myopathy | Palmio *et al.* 2011 | 6 (MRI or CT) | NA | None | N | None | Not reported. | Anterior compartment. |
|  |  |  |  |  |  |  |  |  |
| *DES*-myopathy | Olive *et al.* 2011 | 11 (MRI or CT) | NA | Modified 5-point scale (Fisher et al. 2008) | N | D: DES vs MYOT vs LDB3 vs genetically unconfirmed MFM | Prevalent involvement of ST, S and G compared to AM, BF, SM. The anterior compartment (RF, VL, VI, VM) was relatively spared. | Early involvement of peroneal muscles. Later TA and the posterior compartment muscles (Sol, GM and GL). |
|  | Fischer *et al.* 2008 | 8MRI+11CT | 18-57 | Modified 5-point scale (Fisher et al. 2008) | Y | D: DES vs MYOT vs LDB3 vs FLNC vs CRYAB |  |  |
|  |  |  |  |  |  |  |  |  |
| ABD-  Filaminopathies | Williams *et al*. 2005 | 7 (CT) | NA | None | N | None | Early involvement of SM and ST muscles and later involvement of all hamstring muscles and the adductor magnus.  In more advance stage involvement of quadriceps. | Severe involvement of Sol, asymmetrical involvement of peroneal muscles and a slightly lesser involvement of GM and GL. Sparing anterior compartment and TP. |
|  |  |  |  |  |  |  |  |  |
| Myoshy Myopathy  (MM)  (*DYSF*) | Ten Dam *et al*.2014 | 6 (CT or MRI) | 1-13 | 3 grade scale  0=normal  1=minimal fatty degeneration  2=(nearly) complete fatty degeneration | N | MM vs ANO5 distal myopathy vs LGMD2L | Early involvement of AM. Later SM and VL. In advanced stage there is a progressive involvement of anterior and posterior muscle of thigh with a relatively sparing of BF (short head in one article) and RF.  Spared: S and G until the end stage.  VL presents an early involvement but remains patchy until advanced stages. | Early involvement of GM. Later Sol, GL. In more advanced stage involvement of anterior compartment.  Spared: TP until the end stage. |
|  | Paradas *et al.* 2010 | 14 (MRI) | NA | 4 point scale  0=normal  1=increased signal<50%  2=increased signal>50%  3=100%increased signal | N | D: MM vs LGMD2B vs 1 asymptomatic vs 2 symptomatic carrier |  |  |
|  |  |  |  |  |  |  |  |  |
| *ANO5*-distal  myopathy | Ten Dam *et al.*2014 | 8 (CT or MRI) | 4-28 | See above | N | MM vs ANO5 distal myopathy vs LGMD2L | Early stage not involved. With the progression of the disease involvement of hip adductors, hamstrings and quadriceps muscles.  Spared: S,G (only involved in advanced stage).  RF spared in some reports. | Early involvement of the GM and Sol followed by GL.  Asymmetric muscle involvement could be a feature.  Spared: anterior compartment. |
|  | Mahjneh *et al*. 2012 | 5 (MRI) | 45-73 | 4 point scale:  0=normal  1=hyperintense, patchy but not confluent  2=hyperintense, patchy and confluent involving less than 50% of muscle volume  3=Homogeneously hyperintense; confluent signal involving over 50% of muscle volume | Y | None |  |  |
|  |  |  |  |  |  |  |  |  |
| *GNE*-myopathy | Tasca *et al.* 2012 | 11 MRI+ 2CT | 22-63 | Modified 5-point scale (Fisher et al. 2008) | Y | None | In early stages involvement of BF short head, adductor muscles (AM,AL), hamstring muscles (SM,ST) apart from BF long head that remains relatively spared.  In the anterior compartment RF is often affected, also in the intermediate stages. VM and VI can be involved in the most severely affected patients.  VL is usually spared. | In early stages involvement of TA, EHL, Sol and GM.  GL<GM.  P mild affected in early stages (and always P<TA).  TP often severely affected.  Popliteus least affected muscle. |
|  |  |  |  |  |  |  |  |  |
| *MYH7-*myopathy | Tasca *et al.* 2012 | 5 (MRI) | 40-65 | None | N | None | Not involved in patient with mild clinical course.  In intermediate stage involvement of vastus and adductor longus (±magnus).  In advanced stage all thigh muscles can be involved.  RF affected at very late stage. | TA and EHL most severely affected muscle also in early stage. Gastrocnemius spared also in advanced stage.  Reported calf hypertrophy. |
|  | Muelas *et al.* 2010 | 19 MRI+7 CT | NA | None | N | None |  |  |
|  |  |  |  |  |  |  |  |  |
| *KHLH9*-mutated  distal myopathy | Cirak *et al.* 2010 | 1 MRI | 32 | None | N | None | Fatty infiltration is evident in SM, BF, VI. | Involvement of TA, GM, GL and Sol with relative preservation of TP and peroneus longus. |
| Distal nebulin  Myopathy (*NEB*) | Wallgren-Pettersson *et al*. 2007 | 4 MRI+3CT | 28-64 | None | N | None | Usually not involved. | Selective fatty degeneration of the anterior compartment. |
|  |  |  |  |  |  |  |  |  |
| Distal nebulin  myopathy with  nemaline bodies  (*NEB*) | Lehtokari *et al*. 2011 | 1 MRI+1 CT | 11-61 | None | N | None | Minimal fatty degeneration bilaterally  (in the SM or VI). | Fatty degeneration and atrophy in all lower leg muscles except for TP, which was relatively well preserved. |
|  |  |  |  |  |  |  |  |  |
| *DNM2*-related centronuclear myopathy | Catteruccia *et al.* 2013 | 7 MRI | 8-55 | None | N | None | In early stage not involved.  Involvement begin in BF and SM followed by ST and AM.  The anterior compartment muscles showed moderate to severe involvement in more severely affected patients  Relatively spared RF, S, G. | Different pattern reported.  Fischer *et al.* 2006  Early involvement of GM, GL and Sol. Involvement of the anterior compartment reported in more severely affected patients.  Catteruccia *et al*. 2013  Early involvement of Sol and TA followed by P and GL, GM  Spared: TP. |
|  | Fisher *et al.* 2006 | 10 CT | NA | Modified 5-point scale (Fisher *et al*. 2008) | Y | None |  |  |
|  |  |  |  |  |  |  |  |  |
| HMERF (*TTN*) | Pfeffer *et al.* 2012  ~same cases | 21 (MRI) | NA | 4 point scale indicating severity  From – to +++ | Y | None | ST is the first muscle involved.  In moderate-severe stage involvement of quadriceps femoris, G, S.  Spared: SM, BF. | Peroneus longus is the first muscle involved. Other muscle possibly involved in early stage are TA and EHL.  In advance stage severe involvement of anterior compartment while posterior compartment is affected less frequently and in minor degree. |
|  | Birchall *et al.* 2005  ~same cases | 8 (MRI) | 37-66 | 4 point scale indicating severity  Normal:-; Mild infiltration:+; Moderate infiltration:++; Severe infiltration: +++. | Y | None |  |  |
| IBM | Cox *et al.* 2011 | 32 (MRI) | 68 (mean) | Mildly abnormal <30% of fat compared with muscle.  Moderate abnormal 30-60% of the muscle showed increased signal intensity.  Severely abnormal if at least 60% of the muscle showed increased signal. | N | None | Involvement of quadriceps with relatively sparing of RF.  SM,ST,BF less frequently involved.  Spared: adductor muscles. | All muscles are commonly affected. GM is the most severely affected. |

AM: adductor magnus, BF: Biceps femoris, EHL: extensor hallucis longus, GM: Gastrocnemius medialis, GL: Gastrocnemius lateralis, MFM: myofibrillar myopathy, P: peroneus, RF: rectus femoris, Sol: soleus, SM: semimebranosus, ST: Semitendinosus, TA: tibialis anterior, TP: tibialis posterior, VI: vastus intermedius, VL: vastus lateralis, VM: vastus medialis.

* Fisher 2008 *et al.* included in the literature review despite including only 3 patients as it is the only article reporting the difference between early and late stage.

**
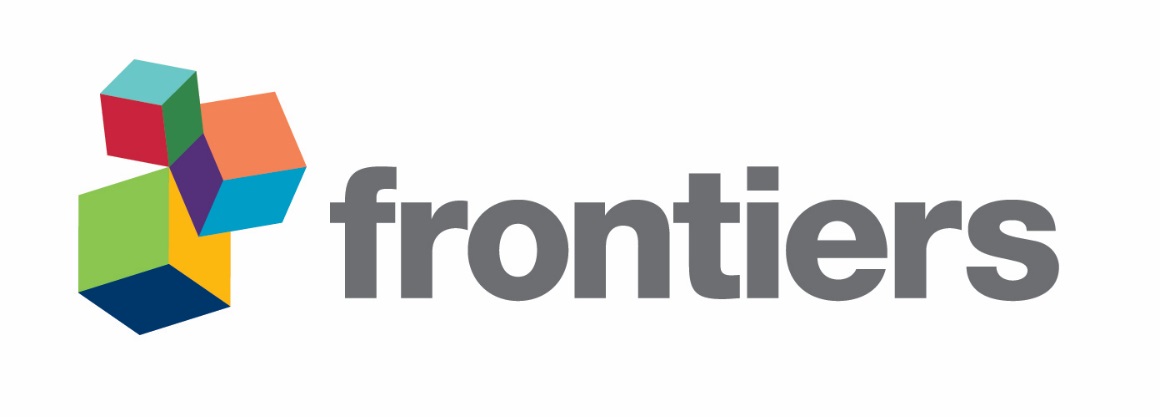
**
